# An *Lmx1a/b* allelic series reveals the role of *Lmx1* genes in cochlear nuclei development

**DOI:** 10.1007/s00441-026-04064-7

**Published:** 2026-04-17

**Authors:** Igor Y. Iskusnykh, Bernd Fritzsch, Ebenezer N. Yamoah, Ekaterina Y. Steshina, Victor V. Chizhikov

**Affiliations:** 1 Department of Anatomy and Neurobiology, The University of Tennessee Health Science Center, Memphis, TN 38163 USA; 2 Department of Neurological Sciences, University of Nebraska Medical Center, Omaha, NE 68198 USA; 3 Department of Translational Neuroscience, College of Medicine-Phoenix, Phoenix, AZ 85004 USA

**Keywords:** Lmx1a, Lmx1b, Cochlear nuclei, Central auditory projections, Inner ear, Roof plate, Rhombic lip, Bmp signaling

## Abstract

*Lmx1a/b* paralogous genes, which arose from the invertebrate *Lmx1b*-like gene, are critical for hearing in multiple vertebrate species, and mutations in these genes cause hearing deficits in humans. While the unique and redundant functions of *Lmx1a/b* in the inner ear are well established, their contribution to the development of the cochlear nuclei, which process and relay auditory information to the brain, is poorly understood. Since cochlear nuclei maturate postnatally, here we analyzed *Lmx1a*^*+/−*^*;Lmx1b*^*+/−*^, *Lmx1a*^*−/−*^, and *Lmx1a*^*−/−*^*;Lmx1b*^*+/−*^ mice that survive past birth. Loss of *Lmx1a* reduced distinct populations of excitatory neurons in dorsal (DCN) and ventral (VCN) cochlear nuclei and their innervation from the inner ear. Additional loss of one *Lmx1b* copy made *Lmx1a*^*−/−*^ phenotypes more severe, revealing that *Lmx1b* acts redundantly with *Lmx1a*. Unlike *Lmx1a*^*−/−*^ mice, excitatory neurons were not affected in *Lmx1a*^*+/−*^*;Lmx1b*^*+/−*^ mice. Thus, while cochlear nuclei are sensitive to *Lmx1a/b* gene dosage, these genes are not completely equivalent, and *Lmx1a* has a more profound role in cochlear nuclei development. *Lmx1a*^*−/−*^ and especially *Lmx1a*^*−/−*^*;Lmx1b*^+/−^ embryos had fewer Atoh1+ progenitors that produce excitatory neurons of the cochlear nuclei, and reduced *Bmp6* expression in the roof plate, the signaling center that induces these progenitors via Bmp signaling. We found that *Lmx1a* is the primary regulator of *Bmp6*, whereas *Lmx1b* contributes only in the absence of *Lmx1a*. Thus, *Lmx1a* plays a major role in the formation of the mature structure and connectivity of both the DCV and VCN, and *Lmx1b* acts redundantly to *Lmx1a* but only partially compensates for *Lmx1a* loss.

**Supplementary Information:**

The online version contains supplementary material available at 10.1007/s00441-026-04064-7.

## Introduction

The inner ear, central projections, and auditory hindbrain nuclei are principal components of the mammalian auditory system, which is essential for sound detection and processing (Driver and Kelley 2020; Pavlinkova 2020; Pyott, et al. 2024; Whitfield 2015). The cochlea of the inner ear, a spiral-shaped organ responsible for the perception of sound, contains the organ of Corti. The organ of Corti harbors mechanosensory inner and outer hair cells and non-sensory supporting cells, which lie in highly specific and asymmetric (tonotopic) order from base to apex of the cochlea (Ceriani, et al. 2025; Kindt and Tarchini 2025; Sun and Liu 2023). From the receptors of the inner ear, signals are transmitted to spiral ganglion neurons in the cochlea, which conduct them via central projections to the cochlear nuclei of the brainstem. Then, neurons of the cochlear nuclei process and relay auditory information to the brain (Macova, et al. 2019; Pyott et al. 2024; Yamoah, et al. 2025).

The inner ear develops and functions through organized sensory regions, stable cochlear synapses that support hearing plasticity, and molecular systems that keep ion balance and sound signal conversion working properly (Friauf and Lohmann 1999; Knipper, et al. 2015; Olaya-Sánchez, et al. 2017; Wilms, et al. 2016). Development of the mammalian auditory system is a complex process that is precisely regulated by multiple genes, including transcription factors. It is believed that gene duplications play a critical role in the origin and evolution of the vertebrate auditory system (Duncan and Fritzsch 2012; Elliott et al. 2023; Fritzsch and Elliott 2017). Paralogous genes arose from a single ancestral gene, often diverge to acquire novel roles during animal development, but can also retain overlapping functions (Chakraborty and Jarvis 2015). Previously, we and others showed that *Lmx1a* and *Lmx1b*, which originated from the invertebrate *Lmx1b*-like gene (Glover et al. 2018), are transcription factors essential for mammalian inner ear formation (Chizhikov et al. 2021). Simultaneous, but not individual, loss of *Lmx1a* and *Lmx1b* in the mouse eliminated the organ of Corti and the spiral ganglion, revealing a redundant role for *Lmx1a/b* during inner ear development (Chizhikov et al. 2021). In addition, less dramatic but significant inner ear abnormalities, including fusion of cochlear and vestibular hair cell domains and defects in the stria vascularis formation and endocochlear potential, were observed in *Lmx1a*^*−/−*^ mice (Koo et al. 2009, Mann et al. 2017, Nichols et al. 2020, Nichols et al. 2008, Renauld et al. 2025), also revealing unique functions for *Lmx1a* during inner ear development that were not compensated by the remaining intact *Lmx1b* gene.

In humans, both dominant and recessive variants of *LMX1A* have been identified in patients with hearing impairment (Lee et al. 2020; Liu et al. 2021; Oziębło et al. 2022; Schrauwen et al. 2018; Wesdorp et al. 2018; Xiao et al. 2023). Haploinsufficiency (an inability of one copy of a gene to produce enough protein to maintain a normal phenotype) was proposed as a pathogenic mechanism underlying *LMX1A*-related autosomal dominant hearing loss (Oziębło et al. 2022). Heterozygous *LMX1B* mutations were associated with hearing loss in a significant fraction of human patients with nail-patella syndrome, a rare genetic disorder characterized by nail and skeletal abnormalities, usually apparent at birth or during early childhood (Bongers et al. 2005). Thus, in addition to mouse studies, human studies also suggest non-redundant, gene-dosage-dependent roles for *LMX1A* and *LMX1B* in hearing maintenance.

The *Lmx1a* and *Lmx1b* genes have been extensively studied in the inner ear, but not in the development of the cochlear nuclei. The cochlear nuclei neurons arise from rhombomeres (r) 2–5 of the hindbrain to form the dorsal (DCN) and ventral (VCN) cochlear nuclei. The cochlear nuclei develop as a unique structure that involves diverse neuronal populations. Specifically, excitatory neurons of the cochlear nuclei arise from the lower rhombic lip, a progenitor domain located adjacent to the hindbrain roof plate. Bmp proteins secreted from the roof plate and its consequent derivative—the choroid plexus, induce Atoh1+ progenitors in the rhombic lip (Alder et al. 1999; Chizhikov et al. 2006b; Hernandez-Miranda et al. 2017; Landsberg et al. 2005; Machold et al. 2007). Atoh1+ progenitors differentiate into excitatory neurons of the cochlear nuclei during an extended period of embryonic development, and produce neurons of several other hindbrain nuclei as well (Butts et al. 2024; Fujiyama et al. 2009; Shepard et al. 2019; Wang et al. 2005).

While cochlear nuclei achieve their mature structure postnatally, *Lmx1b*^*−/−*^ and *Lmx1a*^*−/−*^*;Lmx1b*^*−/−*^ mice do not survive past birth (Mishima et al. 2009). No cochlear nuclei-related defects were reported in *Lmx1a*^*−/−*^ mice. Thus, it remains poorly understood whether *Lmx1a* and *Lmx1b* have unique or redundant roles, including gene-dose-dependent roles, in cochlear nuclei development, or whether their contribution to auditory system development is mainly limited to the inner ear.

In this report, we found that postnatal *Lmx1a*^*−/−*^ mice exhibited profound cochlear nucleus defects, including reduced density of specific excitatory neurons in DCN and VCN, as well as deficits in the innervation of these nuclei by central projections. Interestingly, *Lmx1a*^+/−^*;Lmx1b*^+/−^ heterozygous mice had normal populations of excitatory neurons in cochlear nuclei, indicating a unique role for *Lmx1a* in cochlear nuclei development rather than their dependence simply on *Lmx1a/b* gene dosage (regardless of whether the present *Lmx1* gene copies belong to *Lmx1a* or *Lmx1b*). On the other hand, compared to *Lmx1a*^*−/−*^ mice, the severity of cochlear nuclei phenotypes increased in *Lmx1a*^*−/−*^*;Lmx1b*^+/−^ mice, revealing some functional redundancy between *Lmx1a* and *Lmx1b* during cochlear nuclei development. Cochlear nuclei defects in *Lmx1a*^*−/−*^*;Lmx1b*^+/−^ and *Lmx1a*^*−/−*^ mice were associated with a reduced number of Atoh1+ progenitors and reduced Bmp expression in the roof plate. Combined, our data identified unique and redundant roles for *Lmx1a/b* genes in cochlear nucleus development and in regulating Bmp expression.

## Materials and methods

### Mice

We used *Lmx1a*-null (*Lmx1a*^*drJ*^, Jackson Laboratory strain #000636) (Chizhikov et al. 2006a; Millonig et al. 2000) and *Lmx1b*-null (Chen et al. 1998) mouse alleles. *Lmx1a*^+/−^*;Lmx1b*^+/−^ mice were intercrossed to generate *Lmx1a*^*−/−*^ (*Lmx1a*^*−/−*^*;Lmx1b*^+*/*+^), *Lmx1a*^*−/−*^*;Lmx1b*^+/−^, *Lmx1a*^+/−^*;Lmx1b*^+/−^*,* and wild type mice as previously described. The designated embryonic day 0.5 (e0.5) was the day of a plug. Both male and female mice were used for analysis. All live animal experiments were approved by the University of Tennessee Health Science Center Institutional Animal Care and Use Committee (IACUC; protocols #24–0519, #15–057, and #18–037).

### Lipophilic dye tracing

Mouse heads were fixed in 4% paraformaldehyde (PFA) with 300 mM sucrose for 24 h and then were moved into a fixation solution containing 0.4% PFA and 300 mM sucrose and stored at 4 °C until use (Schmidt and Fritzsch 2019). To trace neuronal projections from the inner ear to the cochlear nuclei, we used NeuroVue dyes (NV Jade, NV Maroon, and NV Red) as previously described. Lipophilic dyes were applied using dye*-*soaked filter strip wedges, which provide a more precise and reliable method of application than dye crystals or injections. Mouse heads were hemi-sected, and the ears were exposed by removing the tympanic membrane. Dye-soaked filter strip wedges were inserted into the apex and into the basal portion of the cochlea near the round window. Dyes were left to diffuse for 4–20 days (depending on the age of the animals) at 37 °C. All mouse samples were processed in parallel, with identical dye placement and corresponding diffusion times. Their brainstems and inner ears were processed, microdissected, and mounted in glycerol.

### Immunohistochemistry

Brains were fixed in 4% PFA in phosphate-buffered saline (PBS) at 4 °C for 12–36 h., washed in PBS, equilibrated in 30% sucrose, and embedded in optimum cutting temperature compound (OCT) as described previously (Chizhikov et al. 2019). Blocks were sectioned on a cryostat at 12 µm. Immunohistochemistry was performed as previously described (Iskusnykh et al. 2023). Briefly, sections were dried at room temperature for 20 min and blocked in PBS containing 0.1% triton-x-100 and 1% normal goat serum. For immunohistochemistry against nuclear markers (Pax6 and Tbr2), antigen retrieval was performed prior to the blocking step by boiling slides in citrate buffer (pH = 6) for 10 min. Then, slides were incubated with primary antibodies diluted in PBS with 1% normal goat serum at 4 °C overnight. Slides were rinsed in PBS, incubated with secondary antibodies for 1 h at room temperature, rinsed in PBS, and mounted in Fluorogel (EMS). Antibodies used were: anti-Calretinin (Rb)—Proteintech (82811–1-RR), diluted 1:1000, anti-Parvalbumin (Ms)—Swant (PV235), diluted 1:1000, anti-Tbr2 (Rat)—eBioscience (cat#14–4875-82), diluted 1:1000, anti-Pax6 (Rb)—Biolegend (Cat#901301), diluted 1:300, anti-activated Caspase 3 (Rb)—Cell Signaling, diluted 1:100, and anti-Atoh1 (Ms)—Developmental studies hybridoma bank, supernatant diluted 1:5. Secondary antibodies were Goat anti-Rat IgG (H + L) Alexa Fluor 488—Invitrogen, Goat anti-Ms IgG (H + L) Alexa Fluor 594 and 488—Invitrogen, Goat anti-Rabbit IgG (H + L) Alexa Fluor 594 and 488—Invitrogen, all diluted 1:200.

### Laser capture microdissection and qRT-PCR

E13 embryos were snap-frozen in OCT, and the hindbrain region was coronally serially sectioned at 10 µm using a cryostat. For consistency, 15 consecutive sections adjacent to the otic vesicles (Fig. 5a) were used to isolate the roof plate (which begins differentiating into the choroid plexus at that stage) by laser capture microdissection using an Arcturus XT LCM machine. RNA was isolated by the Pico Pure RNA purification kit (Arcturus), and cDNA was synthesized using the Bio-Rad cDNA Synthesis Kit. Quantitative real-time PCR (qRT-PCR) was conducted on a Bio-Rad CFX Real-Time PCR System with SYBR Fast qPCR Master Mix, following the manufacturer’s protocol. Gene expression levels were normalized to the housekeeping gene *Gapdh*. Five animals per genotype were analyzed, and three technical replicates were performed for each sample. The following primers were used:

Bmp6 F: atggcaggactggatcattgc; Bmp6 R: ccatcacagtagttggcagcg; (Colucci et al. 2022), and Gapdh F: cgacttcaacagcaactcccactcttcc, Gapdh R: tgggtggtccagggtttcttactcctt (Liu et al. 2009).

### Image acquisition and data analysis

Dye-labeled brainstems were sectioned at 100 µm using a Compresstome microtome and mounted in glycerol. Afterward, the images were obtained using a Zeiss 700 confocal microscope with a 10 × objective (NA 0.45). Z-series stacks were collapsed into single images using the Zeiss Imaging System (Zen 3.8).

To study cochlear nucleus neurons in different genotypes, transverse sections covering the entire hindbrain were collected, with every fifth section stained with cresyl violet to identify the cochlear nuclei. To standardize section positioning along the anterior–posterior (A-P) axis, an equal number of sections were placed on each slide. Sections from comparable A-P levels were compared across different embryos. Three non-adjacent sections (separated by 36–48 µm) containing cochlear nuclei were stained for each antibody marker. Since the size of cochlear nuclei varies even between closely located sections within each mouse, we used cell densities rather than absolute cell counts to compare mice across different genotypes. In sections used for cell counts, all cells positive for specific markers were manually counted in the DCN or VCN, those boundaries were identified using adjacent cresyl violet stained sections. Then, cell densities were calculated by dividing the number of immunopositive cells found within the DCN or VCN region by the DCN or VCN area in a particular section. Since Pax6 and Tbr2 label cell nuclei, and Parvalbumin labels cell bodies, all immunopositive round/oval objects located within the DCN/VCN were counted as positive cells. Since both cell bodies and neuronal processes were highly positive for Calretinin immunostaining, we used DAPI co-labeling to facilitate the identification of individual Calretinin+ cells. A DAPI+ nucleus surrounded by Calretinin+ staining (cell body) was counted as an individual cell (see insets in Fig. 2b-e as examples). Since DAPI labels all cell nuclei regardless of cell type, additional DAPI-stained sections were used to evaluate cell densities in the DCN and VCN. Individual DAPI+ cell nuclei were manually counted. Densities of immunopositive cells (or DAPI+ cell nuclei) in DCN or VCN across different sections from the same animal were averaged to generate a cell density per animal, which was used for statistical analysis (comparisons between different genotypes). Therefore, error bars in figures reflect variations in cell densities between different animals of the same genotype.

Samples from control and mutant mice were co-embedded, co-stained, and imaged using identical camera settings. Fluorescent images were captured with a Zeiss Axio Imager A2 microscope equipped with a Zeiss Axio Cam MRm camera and Axio Vision Rel 4.9 software (Zeiss). Image panels were assembled using Adobe Photoshop.

Statistical analysis of cell counts (multiple group comparison) and qRT-PCR analysis were performed using one-way ANOVA with Tukey’s post hoc test. Quantitative data are presented as mean ± standard deviation, with *p* < 0.05 considered statistically significant.

## Results

### Loss of *Lmx1a* reduces the number of different types of excitatory neurons in postnatal cochlear nuclei, and additional loss of one copy of *Lmx1b* makes the phenotype more severe

**Fig. 1:**
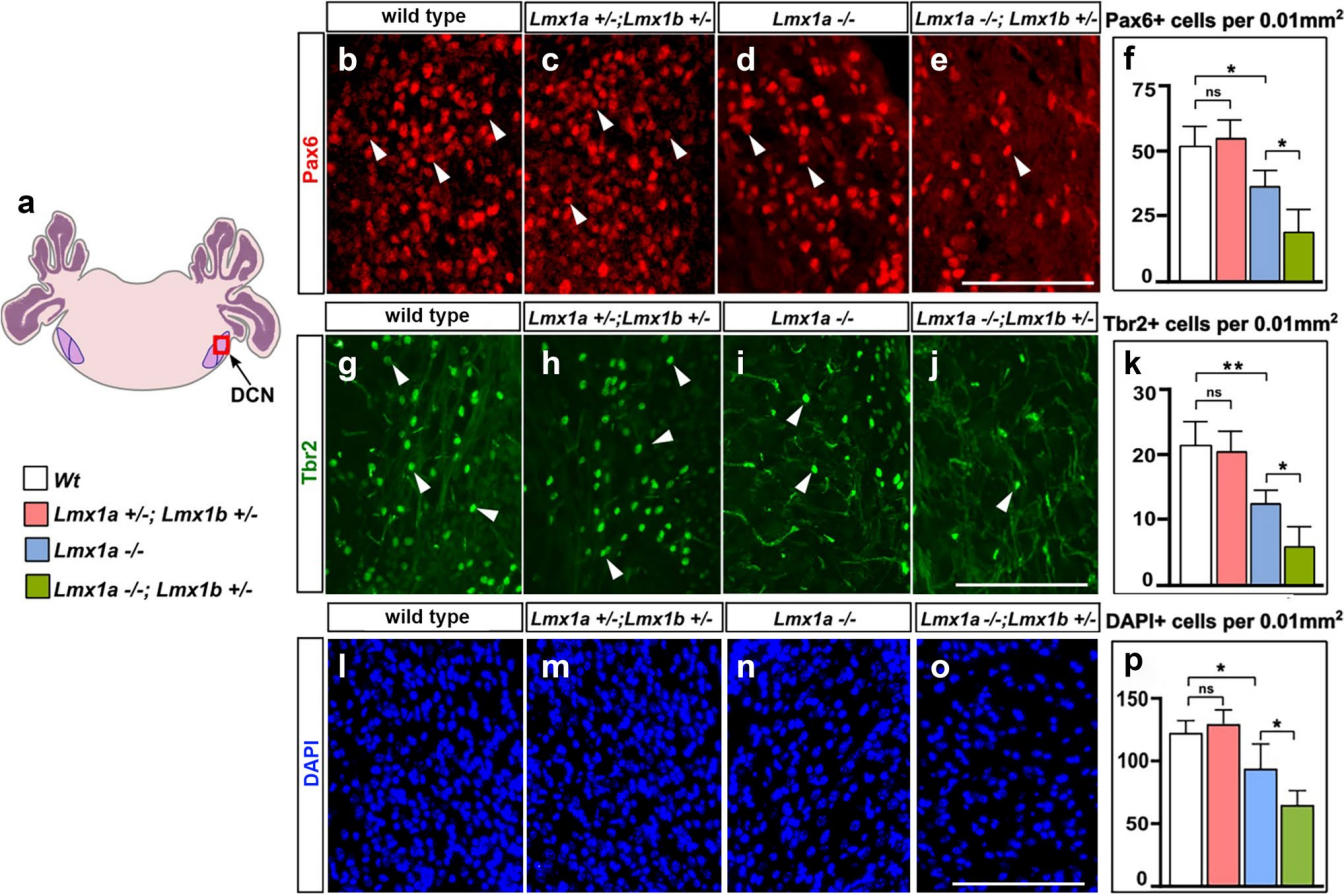
Reduced number of Pax6+ and Tbr2+ neurons in the DCN of *Lmx1a*^*−/−*^ and *Lmx1a*^*−/−*^*;Lmx1b*^+/−^ mice at P14. **a** Schematic representation of the hindbrain. DCN - dorsal cochlear nuclei. Panels **b**-**e**, **g**-**j**, and **l**-**o** show DCN (the region boxed in diagram **a**) stained against Pax6 (**b-e**), Tbr2 (**g-j**) or DAPI (**l-o**). Mouse genotypes are indicated above each panel. **f**, **k**, **p**. Quantification revealed a reduced density of Pax6+ **f** and Tbr2+ **k** neurons, and DAPI+ nuclei **p** in the DCN of *Lmx1a*^*−/−*^ but not *Lmx1a*^+/−^*;Lmx1b*^+/−^ mice compared to wild type mice. Density of both Pax6+ and Tbr2+ cells and DAPI+ nuclei was further reduced in *Lmx1a*^*−/−*^*;Lmx1b*^+/−^ mice compared to *Lmx1a*^*−/−*^ mice. *n* = 4 animals per genotype. **p < 0.01, *p < 0.05, ns – non-significant. Scale bar: 100 µm

To study the role of *Lmx1* genes in the development of cochlear nuclei, we analyzed mice at postnatal day (P) 14, at the onset of hearing, when these nuclei had mostly completed their development (Müller et al. 2019). Since *Lmx1b*^*−/−*^ mice do not survive past birth, *Lmx1a*^*−/−*^*;Lmx1b*^+*/*+^ (referred to as *Lmx1a*^*−/−*^ mice throughout the paper) were compared to *Lmx1a*^*−/−*^*;Lmx1b*^+/−^, *Lmx1a*^+/−^*;Lmx1b*^+/−^*,* and wild type mice. Previous studies identified Pax6 and Tbr2 as markers of excitatory neurons of DCN, specifically granule cells and unipolar brush cells, respectively (Fujiyama et al. 2009). Our immunohistochemical analyses revealed fewer Pax6+ and Tbr2+ neurons in the DCN in *Lmx1a*^*−/−*^ mice relative to wild type controls. In *Lmx1a*^*−/−*^*;Lmx1b*^+/−^ mice, the number of Pax6+ and Tbr2+ cells was further reduced compared to *Lmx1a*^*−/−*^ mice, indicating a more severe phenotype. DAPI nuclear staining also revealed reduced general cell density in the DCN of *Lmx1a*^*−/−*^ and *Lmx1a*^*−/−*^*;Lmx1b*^+/−^ mice, suggesting that markers of excitatory neurons (Pax6 and Tbr2) were not simply downregulated in a subset of DCN cells but that fewer cells populate DCN in these mice (Fig. 1). Interestingly, *Lmx1a*^+/−^*;Lmx1b*^+/−^ mice showed a normal number of Pax6+, Tbr2+, and DAPI+ cells in the DCN (Fig. 1).

**Fig. 2:**
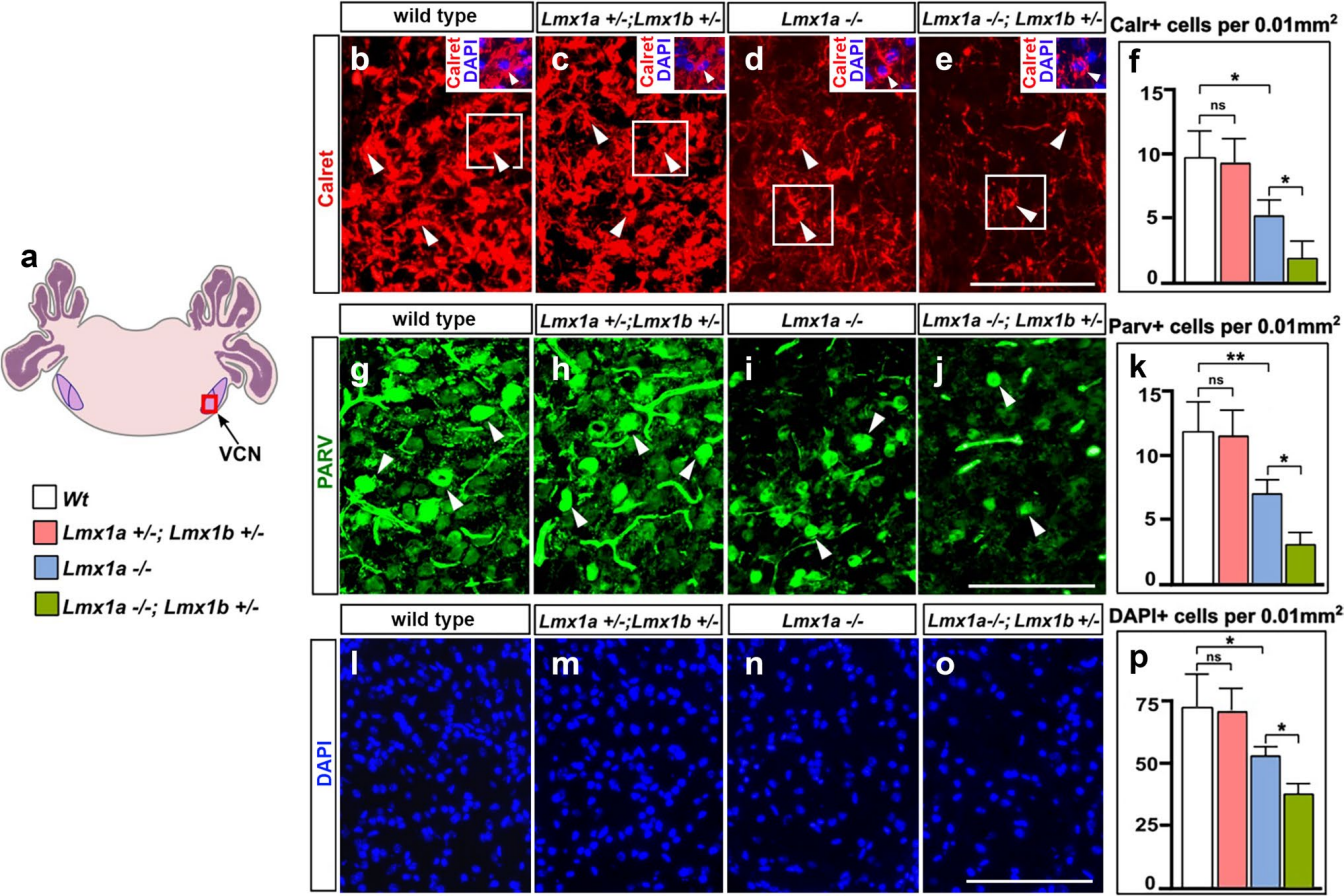
Reduced number of Calretinin + and Parvalbumin + neurons in the VCN of *Lmx1a*^*−/−*^ and *Lmx1a*^*−/−*^*;Lmx1b*^+/−^ mice at P14. **a**. Schematic representation of the hindbrain. VCN - ventral cochlear nuclei. Panels **b**-**e**, **g**-**j**, and **l**-**o** show VCN (the region boxed in diagram **a**) stained against Calretinin **b**-**e**, Parvalbumin **g**-**j** or DAPI **l**-**o**. Insets in **b-e** show boxed regions co-labeling with Calretinin and DAPI to visualize nuclei of Calretinin+ cells. Mouse genotypes are indicated above each panel. **f**, **k**, **p**. Quantification revealed a reduced density of Calretinin+ **f** and Parvalbumin+ **k** neurons, and DAPI+ nuclei **p** in the VCN of *Lmx1a*^*−/−*^ but not *Lmx1a*^+/−^*;Lmx1b*^+/−^ mice compared to wild type mice. Density of both Calretinin+ and Parvalbumin+ cells and DAPI+ nuclei was further reduced in *Lmx1a*^*−/−*^*;Lmx1b*^+/−^ mice compared to *Lmx1a*^*−/−*^ mice. *n* = 4 animals per genotype. **p < 0.01, *p < 0.05, ns – non-significant. Scale bar: 100 µm

Next, we analyzed excitatory neurons in the VCN, using Calretinin and Parvalbumin immunostaining, which label globular bushy and spherical bushy cells, respectively (Bazwinsky-Wutschke and Dehghani 2020; Fujiyama et al. 2009). Loss of *Lmx1a* resulted in a reduction of Calretinin and Parvalbumin positive cells and DAPI+ nuclei in the VCN (Fig. 2). Even fewer such cells were detected in the VCN of *Lmx1a*^*−/−*^*;Lmx1b*^+/−^ mice. In contrast, *Lmx1a*^+/−^*;Lmx1b*^+/−^ mice showed a normal number of Calretinin, Parvalbumin, and DAPI+ cells in the VCN (Fig. 2).

Taken together, our data indicate that the loss of *Lmx1a* alone (in *Lmx1a*^*−/−*^ mice) causes a significant reduction in the number of excitatory neurons in DCN and VCN, suggesting that *Lmx1a* is a major regulator of these neurons. Loss of one copy of *Lmx1b* on an *Lmx1a*^*−/−*^ background leads to a more severe phenotype, indicating that *Lmx1b* partially compensates for *Lmx1a* loss in cochlear nuclei development. Notably, one copy of each *Lmx1* gene (in *Lmx1a*^+/−^*;Lmx1b*^+/−^ mice) was entirely sufficient to maintain the normal number of excitatory neurons in cochlear nuclei.

### Loss of *Lmx1a* alone or together with one allele of *Lmx1b* compromises the expansion of inner ear central projections to cochlear nuclei

Auditory information is transmitted from the inner ear to the central nervous system via spiral ganglion neurons, which send central projection fibers toward the cochlear nuclei. Having observed cochlear nuclei abnormalities in *Lmx1a*^*−/−*^ and *Lmx1a*^*−/−*^*;Lmx1b*^+/−^ mice, we analyzed the effect of *Lmx1* gene dosage on the development of central auditory projections at P2 and e16.5, when these projections form. Dye-soaked filters were inserted into the base or apex of the cochlea in the inner ear, and central auditory projections terminating at the cochlear nuclei were identified. In control (wild type or *Lmx1a*^+/−^*;Lmx1b*^+/−^) mice, central fibers from both the apex and base were typically labeled successfully. Because *Lmx1a* mutant mice had small inner ears, only a fraction of such mutants had both apex and base fibers labeled, while in the remaining, only one type of fiber was labeled. However, combining mice of two different ages enabled us to comprehensively evaluate fibers originating from both the apex and the base in our mutants.

**Fig. 3:**
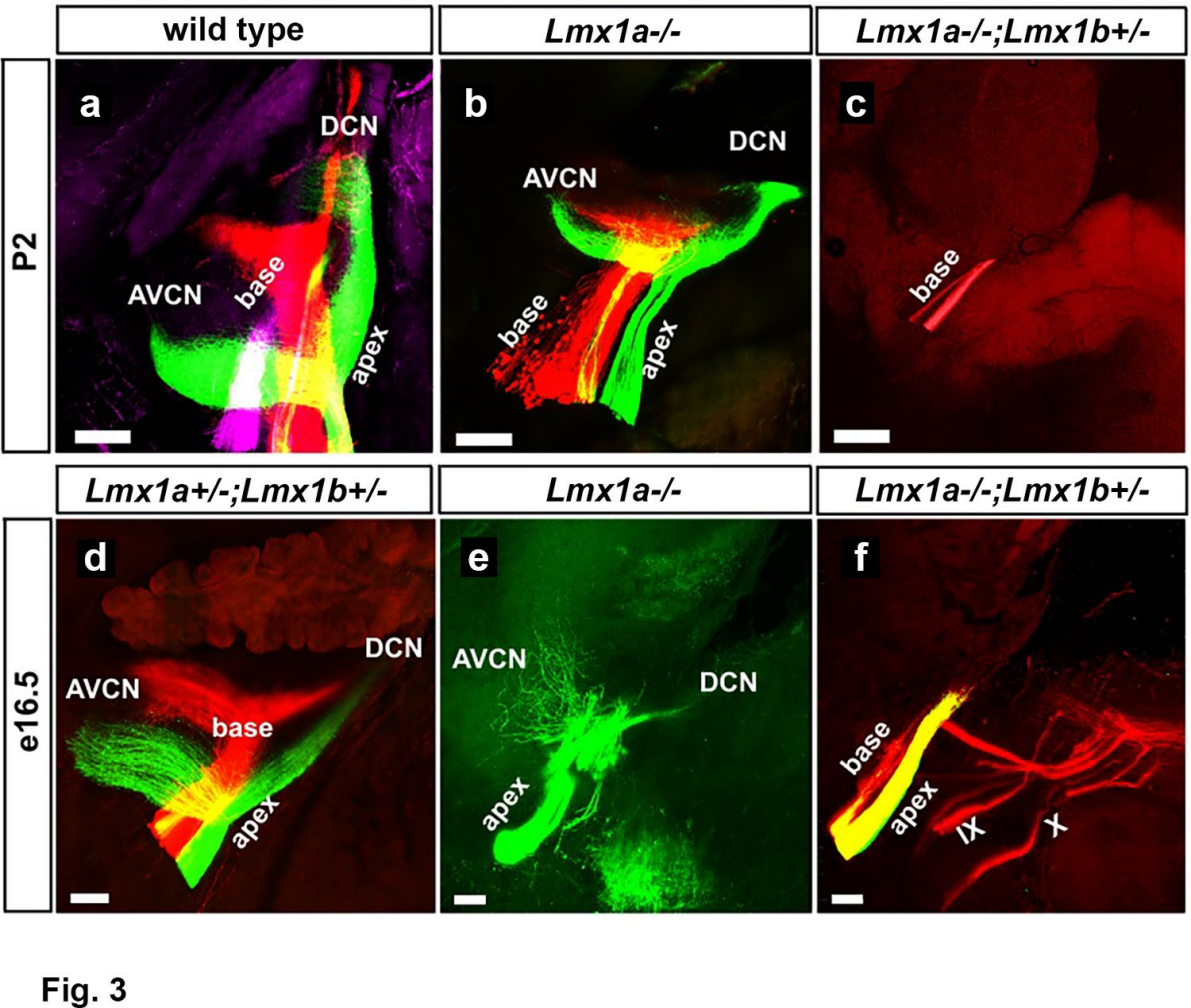
Abnormal central auditory projections in *Lmx1a*^*−/−*^ and *Lmx1a*^*−/−*^*;Lmx1b*^+/−^ mice. Green and red dyes were applied to the apex and base of the cochlea in the inner ear, respectively. Central auditory fibers were traced to the cochlear nuclei in the brainstem. **a**-**f** Control (wild type, **a** or *Lmx1a*^+/−^*;Lmx1b*^+/−^, **d**), *Lmx1a*^*−/−*^ **b**, **e**, and *Lmx1a*^*−/−*^*;Lmx1b*^+/−^ **c**, **f** mice are shown at P2 **a**-**c**, and e16.5 **d**-**f**. AVCN – anterior ventral cochlear nucleus; DCN – dorsal cochlear nucleus. **a**, **d** In control mice, central projections from both the base and apex innervate the DCN and AVCN. **b**, **e** In *Lmx1a*^*−/−*^ mutants, fibers from the apex and the base towards AVCN and DCN were reduced **c**, **f** In *Lmx1a*^*−/−*^*;Lmx1b*^+/−^ animals, branching of the fibers towards both the AVCN and DCN was further reduced compared to even *Lmx1a*^*−/−*^ mutants. Scale bar: 100 μm

In both P2 and e16.5 control (wild type or *Lmx1a*^+/−^*;Lmx1b*^+/−^) mice, central auditory projections from the apex (green) were segregated from the fibers originating at the base of the cochlea (red) as they travel toward the cochlear nuclei (Fig. 3a, d). In *Lmx1a*^*−/−*^ mice, fibers from the base (P2, Fig. 3b) and the apex (P2, Fig. 3b; e16.5, Fig. 3e) towards both the VCN (anterior ventral cochlear nuclei, AVCN) and the DCN were reduced. In addition, fibers from the apex towards the VCN at e16.5 lacked their parallel orientation and were instead widely ramified (Fig. 3e).

In *Lmx1a*^*−/−*^*;Lmx1b*^+/−^ mice, additional loss of projections from the apex and base towards both the VCN and DCN was observed compared to both controls and even *Lmx1a*^*−/−*^ mice (Fig. 3c, f). Thus, reducing *Lmx1a/b* gene dosage results not only in fewer excitatory cochlear nucleus neurons but also in reduced central auditory projections terminating in those nuclei.

### Increased apoptosis at postnatal stages is unlikely to be the primary cause of reduced cochlear neurons in *Lmx1a*^*−/−*^ and *Lmx1a*^*−/−*^*;Lmx1b*^*+/−*^ mice

**Fig. 4:**
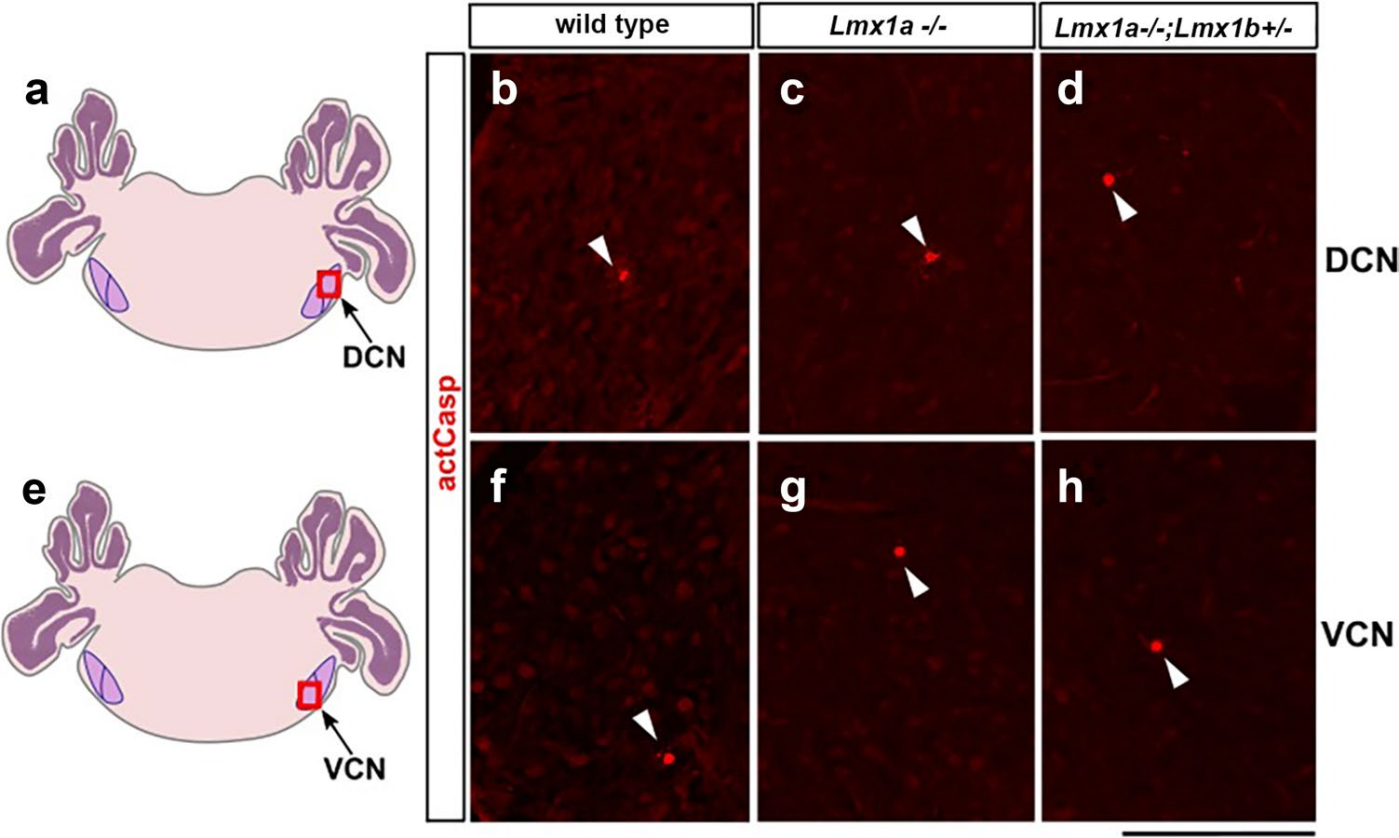
Comparable apoptosis in the DCN and VCN of control, *Lmx1a*^*−/−*^, and *Lmx1a*^*−/−*^*;Lmx1b*^+/−^ mice at P14. **a**, **e**. Schematic representation of the hindbrain. DCN - dorsal cochlear nuclei, VCN - ventral cochlear nuclei. Panels **b**-**d** show DCN (the region boxed in diagram **a**), and panels **f**–**h** (the region boxed in diagram **e**) show the VCN immunostained against an apoptotic marker, activated Caspase 3. Mouse genotypes are indicated above each panel. Arrowheads point to activated Caspase 3-positive (apoptotic) cells. No noticeable difference in apoptosis was detected between wild type, *Lmx1a*^*−/−*^ or *Lmx1a*^*−/−*^*;Lmx1b*^+/−^ mice. Scale bar: 100 µm

Increased cell death could lead to smaller neuronal populations in the postnatal brains of mutant mice (Hollville et al. 2019). To determine if increased apoptosis is involved in the *Lmx1a/b*-dependent disruption of glutamatergic neuronal populations in cochlear nuclei, we performed immunohistochemical staining for activated Caspase 3, a marker of apoptosis (Srinivasan et al. 1998). We found no noticeable difference in apoptosis between wild type, *Lmx1a*^*−/−*^, and *Lmx1a*^*−/−*^*;Lmx1b*^+/−^ mice at P14 (Fig. 4) or P0 (Suppl. Figure 1), suggesting that the observed difference in excitatory neurons in the cochlear nuclei of *Lmx1* mutant mice is largely caused by other mechanisms.

### The roles for *Lmx1a* and *Lmx1b* in the production of Atoh1+ progenitors in the lower rhombic lip and expression of secreted Bmp6 in the roof plate

**Fig. 5:**
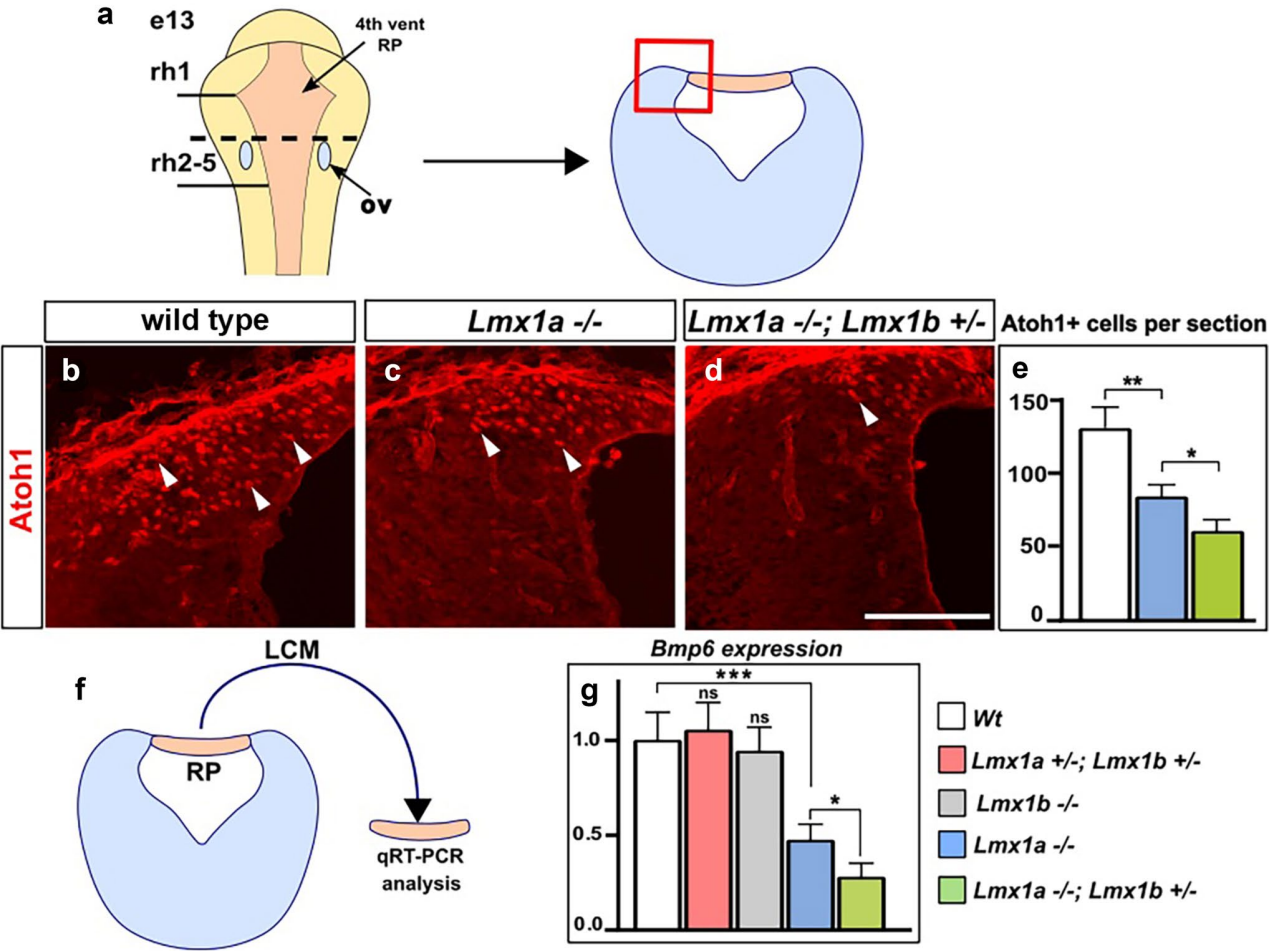
Effect of *Lmx1a* and *Lmx1b* gene dosage on the number of Atoh1+ progenitors in the lower rhombic lip and *Bmp6* expression in the adjacent roof plate. **a** Left. Schematic representation of the whole-mount hindbrain. Hindbrain (4th ventricle) roof plate (4th vent RP) is orange, ov—otic vesicles, rh2-5—rhombomeres 2–5, rh1—rhombomere 1. Right. Schematic of a transverse section at the level of the dashed line. Panels **b**-**d** show the lower rhombic lip region (boxed in the schematic). Panels **b**-**d** show sections immunostained against Atoh1. Mouse genotypes are indicated above each panel. **e** Quantification revealed fewer Atoh1+ cells in the lower rhombic lip of *Lmx1a*^*−/−*^ and *Lmx1a*^*−/−*^*;Lmx1b*^+/−^ embryos than in controls. *n* = 3 animals per genotype. **f** Experimental design. Roof plate (RP) adjacent to rh2-5 rhombic lip was isolated by laser capture microdissection (LCM) and analyzed by qRT-PCR. **g** qRT-PCR analysis of *Bmp6* expression in roof plate of embryos of indicated genotypes. Normalized *Bmp6* expression relative to wild type embryos is shown. *Bmp6* expression was normal in *Lmx1a*^+/−^ *Lmx1b*^+/−^ and *Lmx1b*^*−/−*^ roof plate but was reduced in *Lmx1a*^*−/−*^ roof plate. Additional reduction of *Bmp6* was detected in *Lmx1a*^*−/−*^*;Lmx1b*^+/−^ roof plate compared to *Lmx1a*^*−/−*^ roof plate. *n* = 5 animals per genotype. ***p < 0.001, **p < 0.01, *p < 0.05, ns - non-significant. Scale bar: 100 µm

In the process of cochlear nuclei development, its excitatory neurons arise from Atoh1+ progenitors in rhombomeres 2–5. These progenitors reside in a transient germinal zone that forms adjacent to the hindbrain roof plate—the lower rhombic lip (RL). Our immunohistochemistry revealed a reduced number of Atoh1+ progenitors in the lower rhombic lip of *Lmx1a*^−/−^ mutants at e13, a time point when many excitatory neurons of the cochlear nuclei are born (Shepard et al. 2019). In *Lmx1a*^*−/−*^*;Lmx1b*^+/−^ embryos, the number of Atoh1+ cells was significantly reduced not only compared to wild type controls but also relative to *Lmx1a*^*−/−*^ embryos (Fig. 5a-e), suggesting that early-stage defects in the lower RL progenitor cells caused the reduction of excitatory neurons in the postnatal DCN and VCN.

Atoh1+ progenitors in the rhombic lip are induced by factors secreted from the adjacent roof plate. Such factors include members of the Bmp family, such as Bmp6 (Alder et al. 1999; Chizhikov, et al. 2006b; Landsberg et al. 2005; Machold et al. 2007). *Lmx1a* and *Lmx1b* are co-expressed in the hindbrain roof plate (Iskusnykh et al. 2016; Landsberg et al. 2005; Mishima et al. 2009). Our analysis of JASPAR, an open-access database of manually curated transcription factor binding sites (Rauluseviciute et al. 2024) revealed the existence of 12 putative *Lmx1a* binding sites and seven putative *Lmx1b* binding sites in the 1000 bp region located directly upstream of the mouse *Bmp6* transcription initiation site (Suppl. Figure 2), suggesting that *Lmx1a* and *Lmx1b* may regulate induction of Atoh1+ progenitors via Bmp6.

To determine whether *Lmx1a/b* regulate *Bmp6* expression in a gene-dose-dependent manner, we measured the expression of this gene in the roof plate located adjacent to the lower rhombic lip. Roof plate was isolated by laser capture microdissection from embryos of different genotypes, and *Bmp6* expression was analyzed by qRT-PCR (Fig. 5f). To clarify the role of *Lmx1b* more fully in the regulation of *Bmp6* expression, in addition to *Lmx1a*^*−/−*^, *Lmx1a*^*−/−*^*;Lmx1b*^+/−^, and *Lmx1a*^+/−^*;Lmx1b*^+/−^ embryos, roof plate from *Lmx1b*^*−/−*^ embryos was assayed as well. While no significant difference was observed between wild type, *Lmx1a*^+/−^*;Lmx1b*^+/−^, and *Lmx1b*^*−/−*^ embryos, a significant downregulation of *Bmp6* was observed in *Lmx1a*^*−/−*^ embryos. Even greater *Bmp6* downregulation was observed in *Lmx1a*^*−/−*^*; Lmx1b*^+/−^ embryos (Fig. 5g). Taken together, our data demonstrate that *Lmx1a* plays a major role in promoting *Bmp6* expression and maintaining Atoh1 + progenitors in the lower rhombic lip. *Lmx1b* is a minor regulator of *Bmp6* expression that acts partially redundantly to *Lmx1a* but cannot fully compensate for *Lmx1a* loss.

## Discussion

The evolution of complex vertebrate structures, such as the auditory system, depends heavily on gene duplication and the subsequent functional diversification of paralogous genes (Chakraborty and Jarvis 2015; Duncan and Fritzsch 2012; Elliott, et al. 2021; Fritzsch and Elliott 2017; Shimeld and Holland 2000). Elucidating the unique and redundant functions of paralogous genes is critical to our understanding of vertebrate development and disease. *Lmx1a* and *Lmx1b* genes, originating from the invertebrate *Lmx1b*-like gene, are essential for the adaptability and complexity of several vertebrate systems (Doucet-Beaupré et al. 2015). Previously, in the auditory system, the *Lmx1a/b* genes were mainly studied in the inner ear. Recently, we showed that combined, but not individual, loss of *Lmx1a* and *Lmx1b* eliminated the organ of Corti in the auditory inner ear, the spiral ganglion, and the central auditory projections to the brainstem, revealing largely redundant functions of *Lmx1a/b* in the mouse inner ear development (Chizhikov et al. 2021). Loss of mouse *Lmx1a* alone resulted in milder inner ear defects, which included fusion of cochlear and vestibular hair cell domains, defects in the stria vascularis formation, and the lack of cochlear endolymphatic potential (Huang et al. 2018, Koo et al. 2009, Mann et al., 2017, Nichols et al. 2020, Nichols et al. 2008, Renauld et al. 2025). In zebrafish, *Lmx1b* regulates inner ear semicircular canal morphogenesis (Mori, et al. 2025). In humans, heterozygous mutations in *LMX1A* or *LMX1B* result in hearing deficits (Bongers et al. 2005; Lee et al. 2020, 2022; Oziębło et al. 2022; Schrauwen et al 2018; Wesdorp et al 2018). Together, these studies highlight that in addition to being functionally redundant, *Lmx1a* and *Lmx1b* also exhibit functional activities across species that are not compensated for by the remaining intact copies of the other *Lmx1* gene.

The cochlear nuclei are a central part of the auditory system, which plays a critical role in processing auditory information and relaying it to the brain. The role of *Lmx1a/b* genes in cochlear nuclei development was poorly understood. Since cochlear nuclei maturation is not complete until after birth and *Lmx1b*^*−/−*^ and *Lmx1a*^*−/−*^*;Lmx1b*^*−/−*^ mice do not survive past birth, in the current study we used a wild type, *Lmx1a*^+/−^*;Lmx1b*^+/−^, *Lmx1a*^*−/−*^ and *Lmx1a*^*−/−*^*;Lmx1b*^+/−^ allelic series, to characterize unique and redundant roles of *Lmx1* genes in cochlear nuclei development. Interestingly, we did not observe defects in the cochlear nuclei in *Lmx1a*^+/−^*;Lmx1b*^+/−^ mice. Thus, while heterozygous mutations in any one *LMX1* gene lead to hearing deficits in humans, in the mouse, even a combined loss of one copy of *Lmx1a* and one copy of *Lmx1b* does not compromise cochlear nuclei development. In contrast, loss of two copies of *Lmx1a* gene (in *Lmx1a*^*−/−*^ mice) resulted in a dramatic cochlear nuclei phenotype with nearly 50% reduction in the number of specific excitatory neurons, including granule cells and unipolar brush cells in the DCN and globular bushy and spherical bushy cells in the VCN (Elliott et al. 2023; Jing et al. 2025). This phenotype cannot be explained by simply a reduction of *Lmx1* gene dosage because, in contrast to loss of two *Lmx1a* copies (in *Lmx1a*^*−/−*^ mice), loss of one *Lmx1a* and one *Lmx1b* copy (in *Lmx1a*^+/−^*;Lmx1b*^+/−^ mice) does not lead to a cochlear phenotype. Thus, these genetic experiments suggest a more profound role for *Lmx1a* than *Lmx1b* in cochlear nuclei development. Notably, loss of one copy of *Lmx1b* on an *Lmx1a*^*−/−*^ background further reduced the number of cochlear excitatory neurons. Thus, in the absence of *Lmx1a*, *Lmx1b* can partially support the formation of cochlear nuclei excitatory neurons, revealing a partially redundant role for *Lmx1a* and *Lmx1b* in cochlear nuclei formation.

Previously, it has been shown that *Lmx1a* and *Lmx1b* play pivotal roles in the development of several neuronal populations beyond the cochlear nuclei, such as Cajal-Retzius neurons, midbrain dopaminergic neurons, and glutamatergic (excitatory) neurons in the cerebellum (Andersson et al. 2006, Chizhikov and Iskusnykh 2025, Chizhikov et al. 2010, Iskusnykh et al. 2023, Yan et al. 2011, Yan et al. 2013). Among those types of neurons, the role of *Lmx1a/b* was most extensively characterized in the development of dopaminergic neurons that arise from progenitors in the floor plate of the midbrain. *Lmx1a* and *Lmx1b* genes are co-expressed in the midbrain dopaminergic neuronal progenitors and neurons and regulate multiple steps of their development, including progenitor proliferation, specification, neuronal differentiation, and survival (Chabrat et al. 2017; Deng et al. 2011; Doucet-Beaupré et al. 2016; Laguna et al. 2015; Yan et al. 2011). Interestingly, loss of *Lmx1a* alone leads to a ~ 50% reduction in the number of midbrain dopaminergic neurons, while removing an additional *Lmx1b* copy in the ventral midbrain increases the severity of the phenotype (Yan et al. 2011). Thus, as in cochlear nucleus excitatory neurons, *Lmx1a* is a major regulator of midbrain dopaminergic neuron development. At the same time, *Lmx1b* plays a partially redundant but more limited role, at least during the earlier stages of dopaminergic neuron development.

We did not find a noticeable increase in apoptosis in *Lmx1a*^*−/−*^ and *Lmx1a*^*−/−*^*;Lmx1b*^+/−^ mice at P0 or P14, suggesting that an elevated apoptosis is not the primary cause of the reduced number of excitatory neurons in the cochlear nuclei of these mice. However, we cannot fully exclude a contribution of apoptosis to *Lmx1*-related cochlear phenotypes, since an elevated apoptosis may still occur at stages not investigated in the current paper.

Cochlear nuclei excitatory neurons arise from Atoh1+ progenitors situated in the lower (rhombomeres 2–5) rhombic lip and migrate to form the DCN and VCN (Farago et al. 2006; Fujiyama et al. 2009; Huang et al. 2012). Similar to mature excitatory neurons, the number of Atoh1+ progenitors in the lower rhombic lip decreased in *Lmx1a*^*−/−*^ embryos and was further reduced in *Lmx1a*^*−/−*^*;Lmx1b*^+/−^ embryos. Thus, cochlear nucleus abnormalities in these mice were at least partially caused by the reduction of Atoh1 + progenitors in the rhombic lip during early development. Notably, excitatory neurons were not completely gone in *Lmx1a*^*−/−*^ and *Lmx1a*^*−/−*^*;Lmx1b*^+/−^ mice, suggesting that at least some Atoh1+ progenitors remaining in these mutants were able to differentiate into excitatory neurons that migrate to cochlear nuclei. In contrast, virtually no excitatory neurons were present in the DCN and VCN of *Atoh1*^*−/−*^ mice (Fujiyama et al. 2009; Elliott et al. 2017; Filova et al. 2020), indicating that *Atoh1* is absolutely necessary for rhombic lip progenitors to differentiate into excitatory neurons of cochlear nuclei.

In contrast to progenitors of dopaminergic neurons, which co-express *Lmx1a* and *Lmx1b*, neither of these genes are expressed in the lower rhombic lip. Instead, *Lmx1a* and *Lmx1b* are co-expressed in the hindbrain roof plate (and its later derivative the choroid plexus) located adjacent to the lower rhombic lip in rhombomeres 2–5 (Iskusnykh et al. 2016; Landsberg et al. 2005; Mishima et al. 2009). The roof plate, a critical signaling center in neural development, induces Atoh1+ progenitors in the rhombic lip by secreting Bone Morphogenetic Proteins (Bmps), including Bmp6 (Chizhikov et al. 2006a, b; Liu et al. 2010). JASPAR, an open-access database of manually curated transcription factor binding sites (Rauluseviciute et al. 2024), revealed multiple putative Lmx1a and Lmx1b binding sites in the promoter region of the mouse *Bmp6*, suggesting Bmp6 as a downstream target of *Lmx1a/b* genes. To test the role of *Lmx1a/b* in the regulation of *Bmp6* expression, we isolated the roof plate located adjacent to the lower rhombic lip by laser capture microdissection and assayed gene expression by qRT-PCR. Consistent with the severity of cochlear nuclei phenotypes in our mice, *Lmx1a*^+/−^*;Lmx1b*^+/−^ mice had a normal *Bmp6* expression, while *Lmx1a*^*−/−*^ embryos revealed a lower *Bmp6* expression, which was further reduced in *Lmx1a*^*−/−*^*;Lmx1b*^+/−^ embryos. Reduced *Bmp6* expression in *Lmx1a*^*−/−*^ but not in *Lmx1a*^+/−^*;Lmx1b*^+/−^ and *Lmx1b*^*−/−*^ embryos (Fig. 5f, g) indicates that *Lmx1a* is a major regulator of *Bmp6* in rh2-5 roof plate. *Lmx1b* gene dosage affects *Bmp6* expression only on the *Lmx1a*^*−/−*^ background, indicating that *Lmx1b* plays a partially redundant but more minor role in *Bmp6* expression relative to *Lmx1a*.

In addition to a reduced number of excitatory neurons in the cochlear nuclei, we also observed a reduced innervation of both DCN and VCN by central auditory projections in *Lmx1a*^*−/−*^ and especially *Lmx1a*^*−/−*^*;Lmx1b*^+/−^ mice. Since *Lmx1a* and *Lmx1b* are expressed in the inner ear, we cannot exclude the possibility that inner ear defects contribute to reduced auditory projections in the mice, as mentioned earlier. However, in both *Lmx1a*^*−/−*^ and *Lmx1a*^*−/−*^*;Lmx1b*^+/−^ mice, central auditory projections enter the brainstem but fail to appropriately target the DCN and VCN, suggesting that reduced numbers of excitatory nuclei or deficits in roof plate signaling are more likely causes of these central auditory projection phenotypes. Additional analysis of mice with conditional inactivation of *Lmx1a* and *Lmx1b* in the inner ear and roof plate is needed to define the mechanisms underlying cochlear nuclei innervation defects in these mice.

In conclusion, this report identified a role for the *Lmx1a/b* genes in the development of the mouse cochlear nuclei. Our data indicate that *Lmx1a* is the major regulator of mouse cochlear nuclei development, which is necessary for *Bmp6* expression in the roof plate, induction of Atoh1+ progenitors in the lower rhombic lip, the generation of proper number of different types of excitatory neurons in the DCN and VCN, and proper innervation of these nuclei by auditory central projections from the apex and base of the inner ear cochlea. *Lmx1b* acts at least partially redundant to *Lmx1a* in cochlear nuclei development and can partially support cochlear nuclei formation in the absence of *Lmx1a*.

## Supplementary Information

Below is the link to the electronic supplementary material.

ESM 1 Comparable apoptosis in the DCN and VCN of control, *Lmx1a*^*-/-*^ and *Lmx1a*^*-/-*^;*Lmx1b*^*+/-*^ mice at P0. **A**, **E**. Schematic representation of the hindbrain. DCN - dorsal cochlear nuclei, VCN -ventral cochlear nuclei. Cb – cerebellum. Panels **B**-**D** show DCN (the region boxed in diagram **A**), and panels **F**-**H** show the VCN immunostained against an apoptotic marker, activated Caspase 3. Mouse genotypes are indicated above each panel. Arrowheads point to activated Caspase 3-positive (apoptotic) cells. No noticeable difference in apoptosis was detected between wild type, *Lmx1a*^*-/-*^ or *Lmx1a*^*-/-*^;*Lmx1b*^*+/-*^ mice. Scale bar: 100 µm (PNG 623 KB)

Supplementary file1 (TIF 21119 KB)

ESM 2 Putative Lmx1a/b binding sites in the mouse *Bmp6* upstream region. The blue box shows exon 1 of the mouse *Bmp6* gene. Grey boxes show putative Lmx1a and Lmx1b binding sites upstream of *Bmp6* predicted by JASPAR with *p*<0.01 (PNG 20.6 MB)

Supplementary file2 (TIF 21118 KB)

## Data Availability

Data are provided within the manuscript or supplementary information files.
