# Supplementary figures and images for: An Lmx1a/b allelic series reveals the role of Lmx1 genes in cochlear nuclei development
Source: Cell Tissue Res. 2026 Apr 17;404(1):4. doi: 10.1007/s00441-026-04064-7 (PMC13086785; doi:10.1007/s00441-026-04064-7)

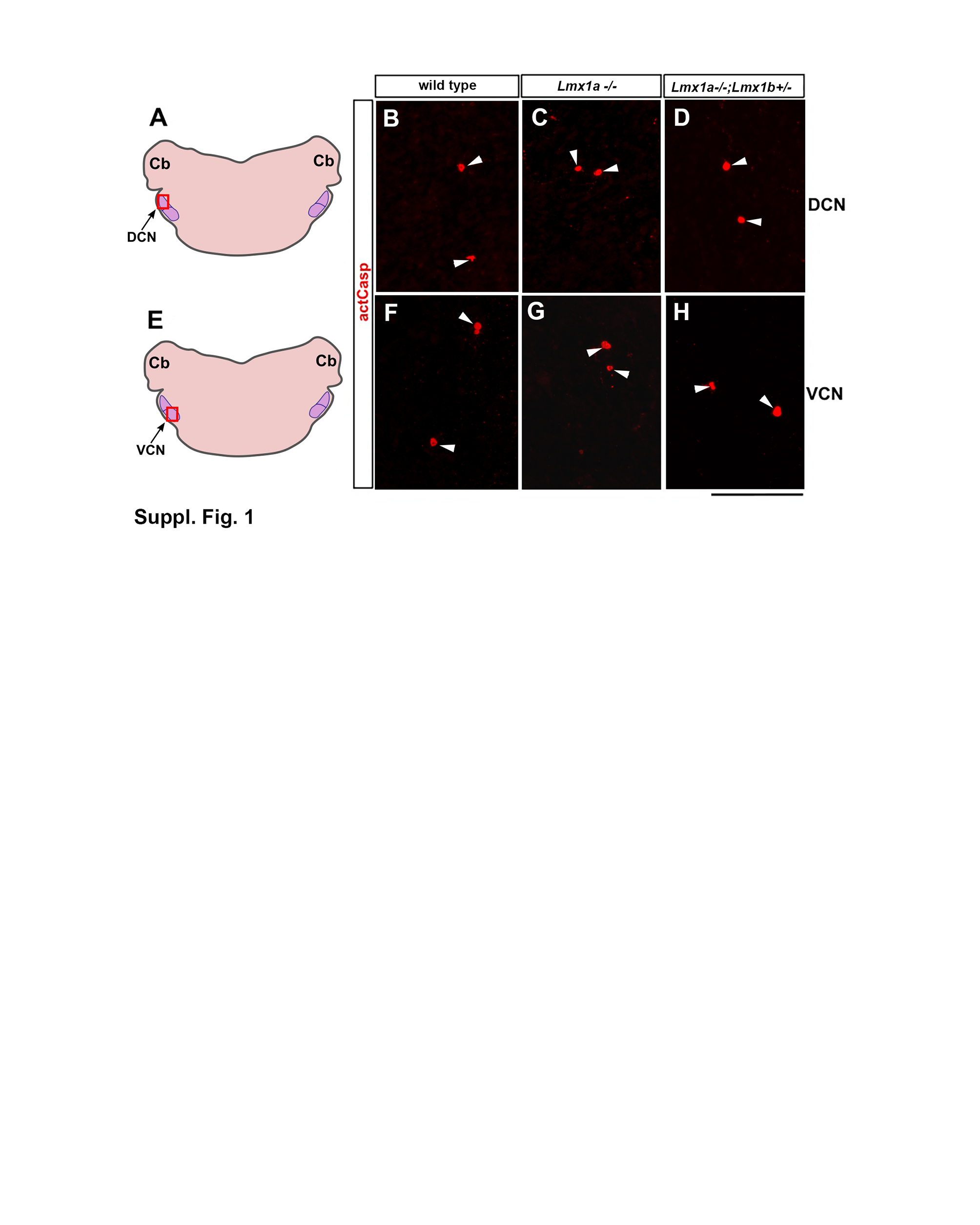

Supplement: Supplementary file 1 — Comparable apoptosis in the DCN and VCN of control, Lmx1a-/- and Lmx1a-/-;Lmx1b+/- mice at P0. A, E. Schematic representation of the hindbrain. DCN - dorsal cochlear nuclei, VCN -ventral cochlear nuclei. Cb – cerebellum. Panels B-D show DCN (the region boxed in diagram A), and panels F-H show the VCN immunostained against an apoptotic marker, activated Caspase 3. Mouse genotypes are indicated above each panel. Arrowheads point to activated Caspase 3-positive (apoptotic) cells. No noticeable difference in apoptosis was detected between wild type, Lmx1a-/- or Lmx1a-/-;Lmx1b+/- mice. Scale bar: 100 µm (PNG 623 KB) [file 441_2026_4064_Fig6_ESM.png]

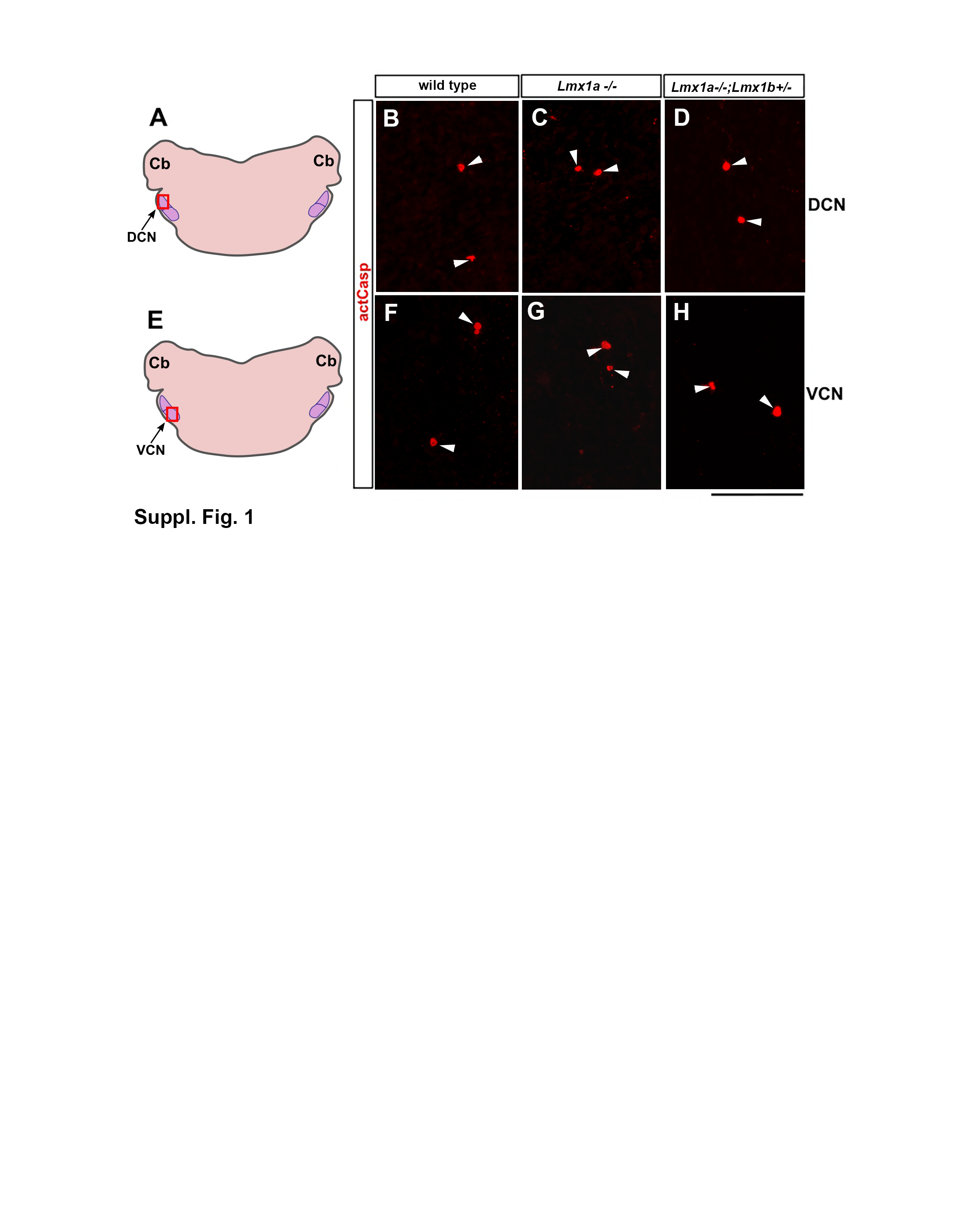

Supplement: Supplementary file 2 — Supplementary file1 (TIF 21119 KB) [file 441_2026_4064_MOESM1_ESM.tif]

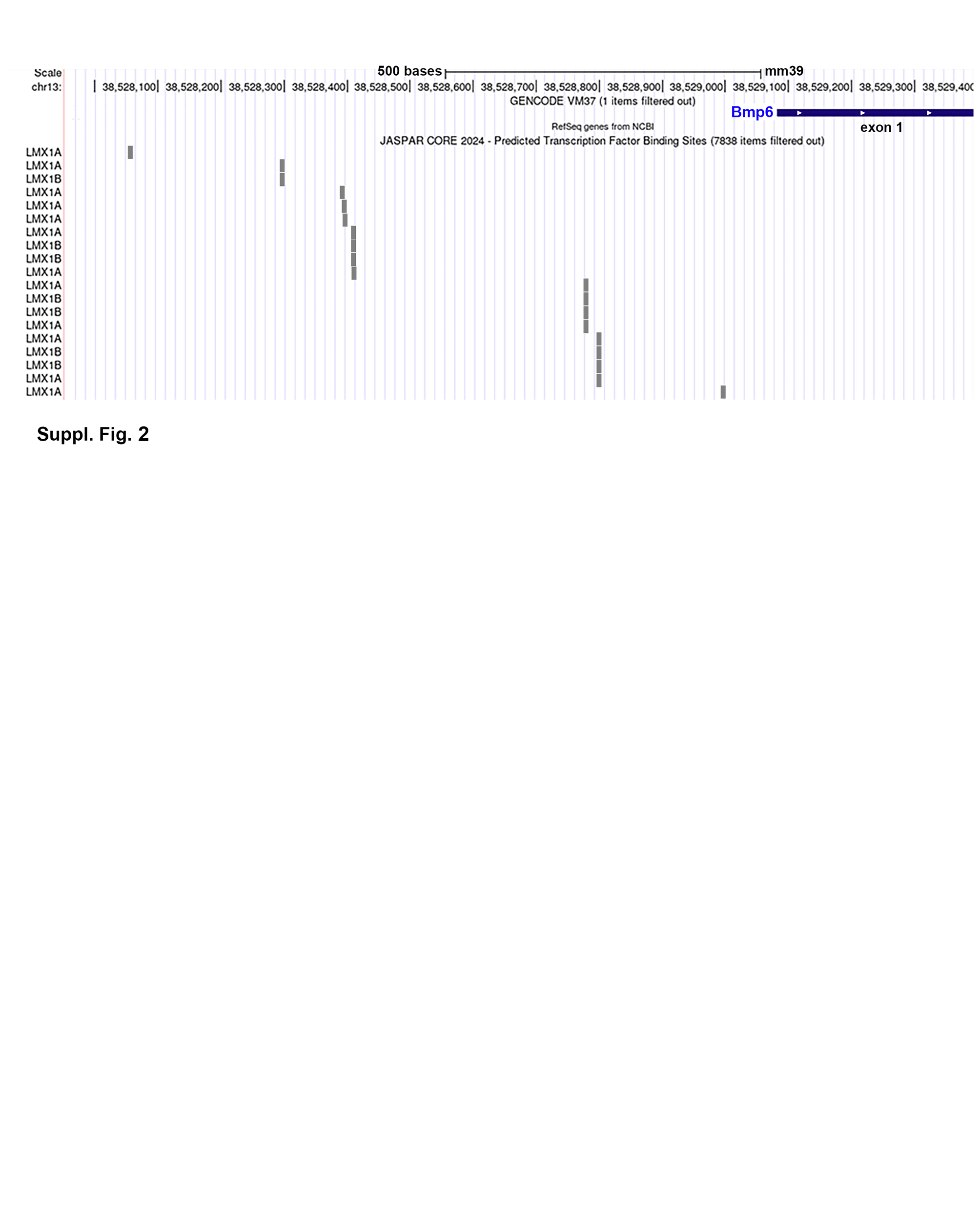

Supplement: Supplementary file 3 — Putative Lmx1a/b binding sites in the mouse Bmp6 upstream region. The blue box shows exon 1 of the mouse Bmp6 gene. Grey boxes show putative Lmx1a and Lmx1b binding sites upstream of Bmp6 predicted by JASPAR with p<0.01 (PNG 20.6 MB) [file 441_2026_4064_Fig7_ESM.png]

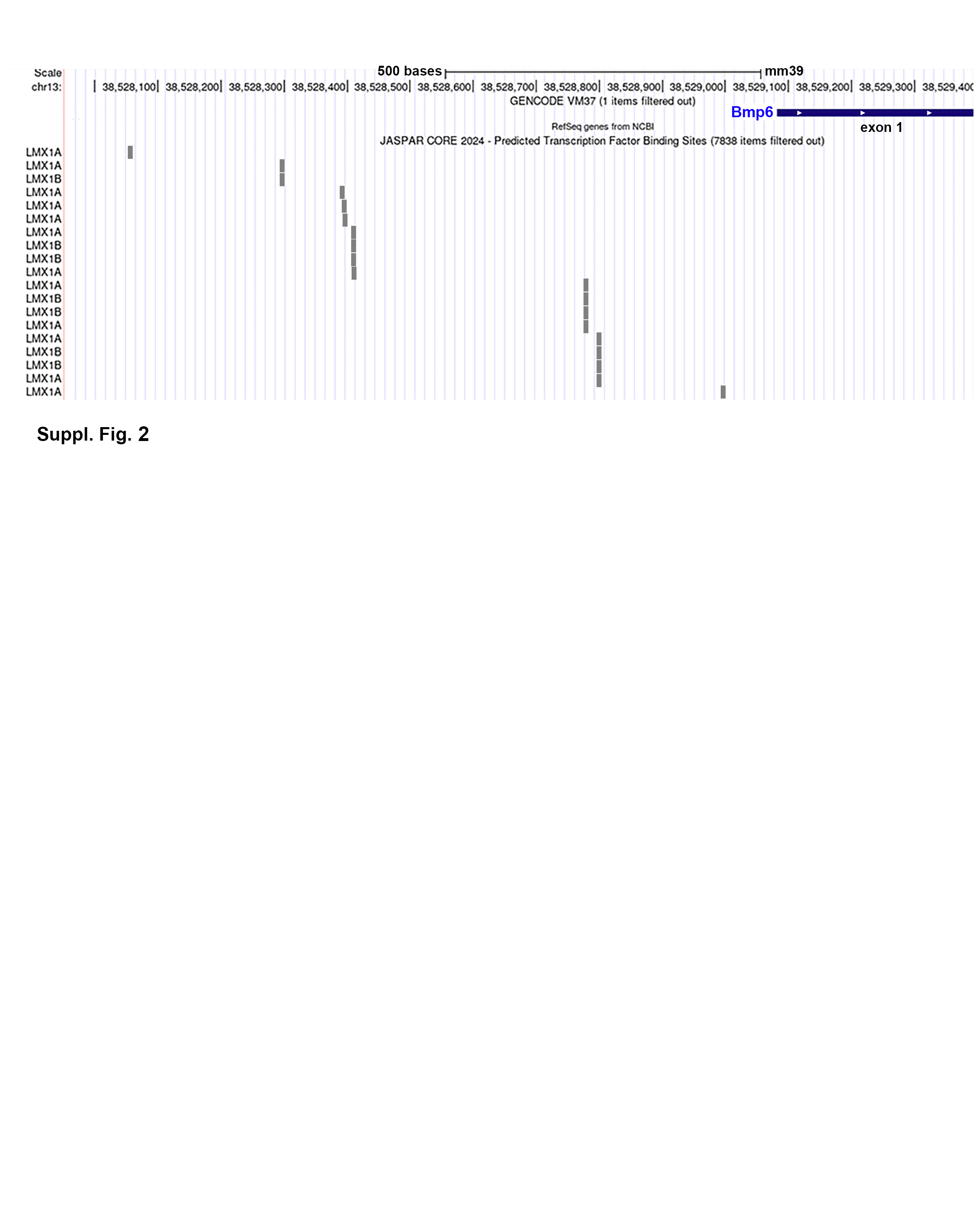

Supplement: Supplementary file 4 — Supplementary file2 (TIF 21118 KB) [file 441_2026_4064_MOESM2_ESM.tif]
